# Investigating Students' Perceptions of Online Learning Use as a Digital Tool for Educational Sustainability During the COVID-19 Pandemic

**DOI:** 10.3389/fpsyg.2022.886272

**Published:** 2022-06-21

**Authors:** Mohammed Ayid Alqahtani, Mahdi Mohammed Alamri, Amer Mutrik Sayaf, Waleed Mugahed Al-Rahmi

**Affiliations:** ^1^Educational Technology Department, College of Education, University of Bisha, Bisha, Saudi Arabia; ^2^Educational Technologies, King Faisal University, Al Hofuf, Saudi Arabia; ^3^Faculty of Social Sciences and Humanities, School of Education, Universiti Teknologi Malaysia, Skudai, Malaysia

**Keywords:** information system (IS) success model, sustainability, COVID-19 pandemic, e-learning system, structural equations modeling (SEM)

## Abstract

Previous research on e-learning in underdeveloped countries has seldom taken a comprehensive approach. A literature review of recent published research in the field of e-learning use during the COVID-19 epidemic is also included in this study. Therefore, the aim of this study is to look at the technology acceptance model (TAM) and information systems (IS) performance models to see how system quality (SYQ), service quality (SEQ), and quality of life (QoL) are related, as well as the mediating impact of perceived ease of use (PEU) and perceived usefulness (PU), affect students' behavioral intention to use (BIU), and actual use of an e-learning system (AUE) as sustainability for education during the COVID-19 pandemic. Path analysis and structural equation modeling (SEM) were used to evaluate the research model, using the data from e-learning users obtained through a survey. Participants were e-learning users from two Saudi Arabian public universities. The findings revealed that PU and ease of use were positively correlated and influenced by SYQ, SEQ, and QoL in education, and that PEU and PU were positively influenced by students' BIU and AUE system. In the sense of e-learning in developing countries, previous studies rarely looked at an integrated model. This paper also attempts to provide a recently published study in the area of the use of an e-learning system as sustainability for education during the COVID-19 pandemic. There is a lot of ongoing research.

## Introduction

Education is essential for both individual growth and community sustainability. Several educational institutions began to move to online teaching during the COVID-19 outbreak to maintain continuous and effective instruction (Sahu, 2020). Students' views of online learning and their excitement for study should be evaluated for long-term online learning as online classes may substitute classroom learning for a long time. In addition, the COVID-19 outbreak has ushered in a new era of education. Even when COVID-19 is over, we may expect more online educational opportunities to emerge. Many courses at all levels of education have been pushed to switch from traditional classroom instruction to online learning (UNESCO, 2021). On the other hand, the majority of teaching faculties have no prior experience in online teaching and are unaware with the technological tools that must be used to provide online lectures (Scarborough, 2021). Furthermore, many educational institutions may lack the requisite information technology to facilitate online training, such as virtual classroom software (Mirzajani et al., 2016).

According to some academics, online teaching is analogous to classroom teaching, and the role of an online teacher is similar to that of a classroom faculty member (Wray et al., 2008). The COVID-19 pandemic has transformed higher education. As a result, there are several opportunities to learn from the educational accomplishments of other institutions to better our collective approach to COVID-19 now and in the future. Furthermore, COVID-19 had a negative impact on student wellbeing in four countries: Cambodia, Nigeria, Oman, and Spain, causing us to look at the cross-cultural effects of COVID-19 on higher education students in Saudi Arabia.

Through the construction of an effective knowledge flow inside enterprises, e-learning systems give solutions to disseminate knowledge and information, facilitate learning, and increase outcomes (Menolli et al., 2020). Every human being may gain the information, skills, attitudes, and values required to construct a sustainable future in school through education for sustainable development. Education for sustainable development is all about incorporating major sustainable development challenges into teaching and learning (UNESCO, 2014). It also necessitates interactive teaching and learning approaches that encourage and empower students to modify their behavior and take action in the interest of long-term sustainability. As a result, e-learning for sustainable development improves skills such as critical thinking, imagining future possibilities, and joint decision-making (UNESCO, 2014). E-learning platforms, such as Canvas, Blackboard, and Moodle, are popular. Learning management systems (LMS) are enabled by these platforms. Students, employees, administrators, instructors, organizations, and other participants benefit from such systems as they aid and improve learning processes while facilitating efficient information flow (Garavan et al., 2019). Instructors and administrators can utilize features such as producing modules to arrange material and learning resources for mini-courses, or networking networks such as chats, forums, and video exchange to build suitable training and skills sharing (Wang et al., 2011). In recent years, the use of diverse e-learning capabilities to increase organizational and workplace learning has become a commodity. Education and training, human resources development, and corporate training are all terms used in the literature to describe training or knowledge production: Garavan et al. are examples of workplace learning (Garavan et al., 2019). E-learning is described as the use of technology as a learning mediation tool that allows users to quickly acquire knowledge and interact with others through the internet (Wu et al., 2012). Online learning include computer-assisted learning, e-learning as a source of sustainability in higher education, remote learning, and online learning (Ho and Dzeng, 2010). To improve the interaction between students, teachers, and the course, online learning is conducted *via* the internet or intranet. The feasibility of e-learning in higher education is based on fostering an equitable partnership between students and teachers, allowing them to pool resources and collaborate to achieve greater success (Shipee and Keengwee, 2014) and better meet the basic educational goal of enhancing learning effectiveness and performance. As a result, students' perceptions of e-learning technology are critical, and they must be addressed before these technologies can be fully integrated into education (Ozdamli and Uzunboylu, 2014). Researchers should investigate learners' perceptions of e-learning because it provides an advantage to real educational institutions, such as schools, colleges, and universities, as well as organizations, by allowing a better understanding of key factors that influence the intentions and use of e-learning as a source of educational sustainability (Mohammadi, 2015; Al-Rahmi et al., 2019, 2020). In recent years, there has been much discussion on the relationship between the use of e-learning as a source of sustainability in higher education and corporate learning (Khandakar and Pangil, 2019; Turi et al., 2019; Xiang et al., 2020). However, there is a lack of systematic work that combines and conceptualizes the findings to help universities move from information- to knowledge-based businesses (El Kadiri et al., 2016). From virtual reality (VR) settings (Costello and McNaughton, 2018) to mobile computing apps (Renner et al., 2020) to learning analytics and adaptive learning (Zhang et al., 2019), recent technological advancements have led to an increase in research that enhances organizational learning through the use of e-learning as a sustainability strategy in higher education. College affordability, which impacts students, would grow when lifetime learning in educational institutions is supported by e-learning systems that combine self-learning methods and the integration of personalized cloud storage. To define a sustainable e-learning system, we must first identify and assess the factors and educational sustainability needs. Much research has been done on the long-term survivability of e-learning systems. However, they usually concentrate on just one aspect of long-term viability. Individual features, for example, have been studied by many academics (Kruchten, 2015), while economic factors (Downes, 2007; Koohang and Harman, 2007), and social dimensions (Littlejohn, 2003) have been studied by others. The environmental element of e-learning system sustainability was explored by Dong et al. (2009) and Roy et al. (2008). Students and innovative pedagogies that bring them closer to the social reality and its core conflicts are the focus of attention in sustainable education, according to Alcalá del Olmo and Gutiérrez Sánchez (2019). The goal is for students to have a better grasp of their e-learning environment as a result. The University should be a leading force in educating students about sustainability and the changes it necessitates. As a result, focusing on values that contribute to critical thinking and include sustainability issues in the content of subject materials *via* e-learning is a priority in education (Alcalá del Olmo and Gutiérrez Sánchez, 2019). In digital learning environments, students must also be able to engage directly in innovations, experiments, and shared experiences. All these activities are carried out with the help of modern e-learning techniques (Lee and de Vries, 2019). As a result, the success of an e-learning system is considered as the success of an information system (IS) and research on the technology acceptance model (TAM) adoption has resulted in a variety of complementary and opposing models for the adoption of studies, most of which are linked to the adoption of IS and the use of e-learning as a long-term solution in higher education. Therefore, Azeiteiro (2015) revealed that education in sustainability through e-learning might be particularly significant for sustainable development in a research conducted in a Portuguese University. However, the authors discovered that some students had troubles in acquiring certain competencies, indicating that further studies are needed to find the optimal instructional paradigm. In Spain and the USA, Racovita-Szilagyi et al. (2010) performed e-learning experiments with 400 University teachers in the field of social work. The authors sought to discover how they felt about the possibilities of e-learning in their classrooms. Regardless of the scope or emphasis of the project, e-learning efforts face a similar difficulty in the tertiary sector (Gunn, 2010). The Saudi Ministry of Education announced remote learning for public and private schools and colleges on 8 March 2020, as a preventative and precautionary step to stop the spread of the coronavirus. It is crucial to express the first impressions of the abrupt switch to an e-learning system, as this will serve as a benchmark for future improvements. The present study's goals are to look at the students' e-learning experiences at the BU and KFU in Saudi Arabia, as well as their thoughts on the benefits and drawbacks of e-learning, as well as their suggestions for enhancing e-learning. However, *via* an inquiry of students' perceptions on e-learning systems, this research gives the high-level sustainability elements of e-learning systems: a merger of the technological acceptance model and the IS success model as sustainability in higher education. As a result, the research question is: What are the factors that contribute to the effective use of an e-learning system as a long-term educational resource? To answer this question, the researchers looked into and established a new model based on a combination of TAM and IS success models for using e-learning systems as a resource for educational sustainability.

## Research Model and Hypotheses Development

**Figure 1:**
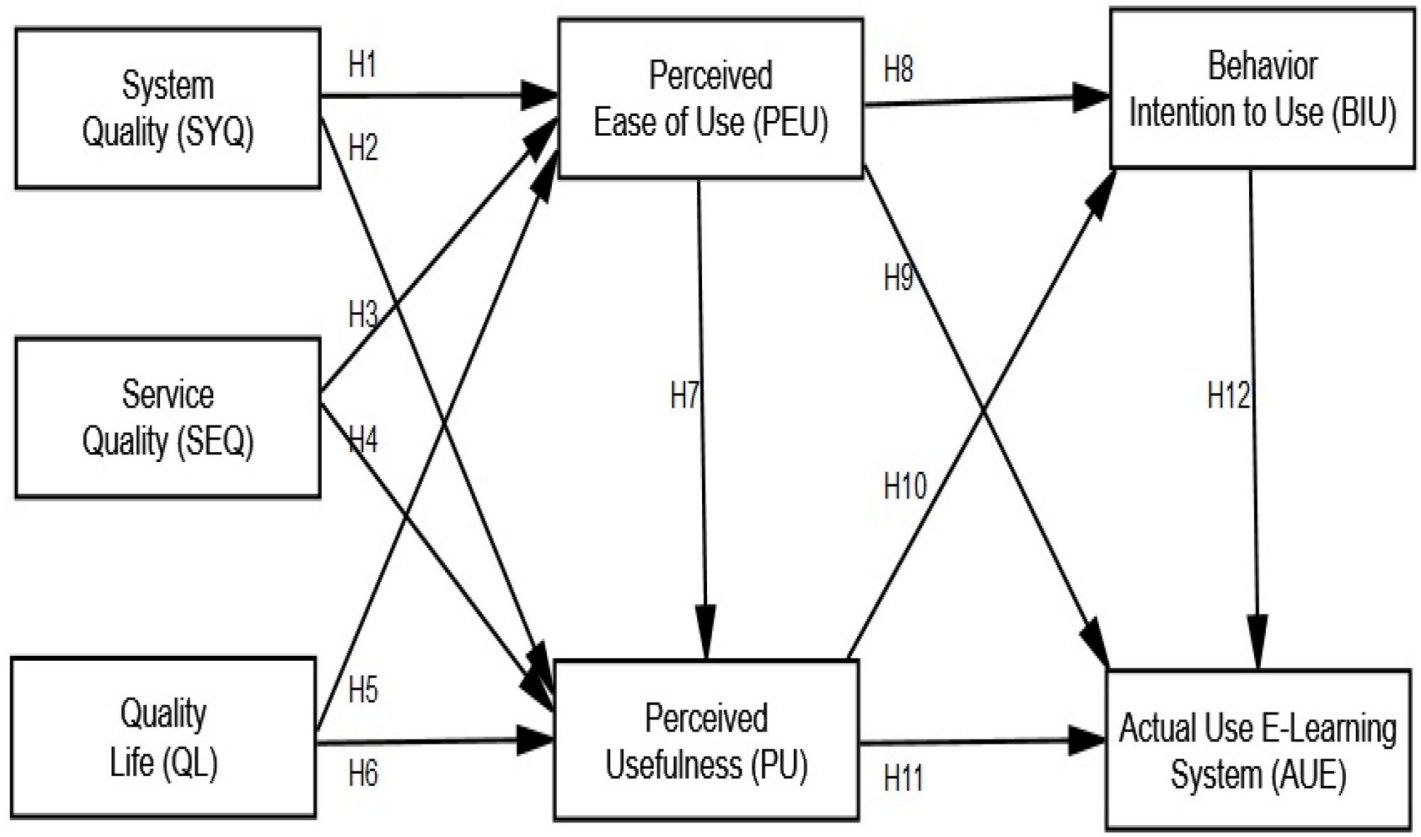
Research model.

E-learning is the most extensively employed methodology for accessing resources *via* computers, laptops, cellphones, and tablets. Technology has a significant advantage in education and the teaching–learning environment. Compared to traditional learning techniques, e-learning offers more access to learning resources, faster communication, and academic collaboration. It has been challenging to come up with a clear definition of e-learning due to ongoing technological advancements. Many studies have attempted to define e-learning in a variety of ways. Some studies (Cidral, 2018) defined e-learning as the use of technology for the learning process, while others (Eom et al., 2012) defined it as an IS capable of absorbing a variety of educational resources *via* email, discussion, assignments, quizzes, and live chat sessions. As a result, we shall use e-learning as an IS in Figure 1. As a result, the success of an e-learning system is considered as the success of an IS. As a way to ensure the sustainability of education, the e-learning system has altered higher education teaching and learning. Davis (1989) developed TAM to explain IS/IT adoption, and highlighted two key assumptions that drive IS adoption: perceived benefit and perceived ease of use (PEU). As a result, the study's main focus is on perceived utility and ease of use acceptance. The behavior of students in e-learning environments is studied using constructivist technology adoption methodologies. TAM (Davis, 1989) and the IS performance model (DeLone and McLean, 2003) are two of the most well-known theoretical contributions to adoption analysis, and they are frequently used by researchers to use e-learning systems as a source of educational sustainability during the COVID-19 pandemic. System quality (SYQ), service quality (SEQ), quality of life (QoL), PEU, perceived usefulness (PU), behavioral intention to use (BIU), and actual use of an e-learning (AUE) were investigated in the current study (Figure 1) to see if e-learning might be employed as a long-term solution for education during the COVID-19 epidemic in higher education.

### System Quality

System quality (DeLone and McLean, 2003) describes the organizational structure, responsibilities, procedures, processes, and resources used to achieve quality management. SYQ also refers to the technological performance, as well as the accuracy and efficiency of the information-producing communication system, according to the IS performance model established by DeLone and McLean (2003). In reality, it is connected to the presence or absence of a problem in the system and incorporates the required properties and metrics of an IS (DeLone and McLean, 2003). The quality of e-learning systems has been shown to have a considerable beneficial influence on satisfaction with education (Alsabswy et al., 2013), and Tajuddin et al. (2013) and Rapley (2003) identified a link between satisfaction with learning and the blended learning system's SYQ. E-learning systems are also expected to be a long-term educational option (Cheng et al., 2012; Alam et al., 2021). As a consequence, we projected that SYQ would increase individual satisfaction and system use intentions. As a result, the following hypotheses emerged from this research.

Hypothesis (H1): SYQ and PEU have a substantial link.
Hypothesis (H2): SYQ and PU have a substantial link.

### Service Quality

The assessment of a customer's service expectations in relation to the performance of an or-overall generation's performance (DeLone and McLean, 2003). SEQ refers to the degree of service provided by e-learning systems (Wang and Wang, 2009), training (Petter et al., 2008), and student learning. Despite the fact that the e-learning system is a subordinate to SYQ in the model, some academics argue that, given the rapid changing positions in e-learning systems in recent years, it might stand alone as an independent variable (Wang and Liao, 2008). SEQ has been demonstrated in several studies to have a positive impact on e-learning consumption and a positive impact on satisfaction (Cheng et al., 2012; Tajuddin et al., 2013). In addition to the long-term educational purpose of adopting an e-learning system (Poulova and Simonova, 2014; Xu et al., 2014), in this investigation, SEQ is anticipated to have a favorable impact on both person satisfaction and intention to utilize it. The investigation demonstrated the empirical importance of the relationship between SEQ and PU stated in the conceptual model of Hagos et al. (2016). As a result, the following hypotheses emerged from this study.

Hypothesis (H3): There is a strong link between SEQ and PEU.
Hypothesis (H4): There is a strong link between SEQ and PU.

### Quality of Life

The widespread use of the term “quality of life” (QoL) in a variety of settings and for a variety of purposes by academics in many professions makes it somewhat problematic (Rapley, 2003; Lwoga, 2014). Rapley (2003) examines a number of different QoL definitions at different levels of aggregation. At the individual level, he thinks that Robert Cummins' concept of QoL is the most significant (and operationalized by the comprehensive QoL scale). When determining the QoL, Cummins (Phillips, 2006) examines both subjective and objective aspects of schooling. The output and QoL success aspects (Petter et al., 2008) explain the optimal characteristics of the performance of an e-learning system. One example is the information that students will gain as a result of using the e-learning system to ensure educational sustainability. As a result, it includes indicators of the system's ability to provide high-quality information and its usefulness in terms of user satisfaction (Cummins, 1997; Rapley, 2003; Kim et al., 2012), as well as the intention to use an e-learning system as a long-term educational solution (Rapley, 2003; Xu et al., 2014). As a result, in this study, it is anticipated that content and QoL have a positive influence on people's contentment and usage intentions. As a result, the following hypotheses emerged from this study.

Hypothesis (H5): There is a strong link between QoL and PEU.
Hypothesis (H6): There is a strong link between QoL and PU.

### Perceived Ease of Use

Perceived ease of use is described as a person's belief that the use of a system would be painless (Davis, 1989), and it is a significant factor in the adoption of revolutionary technological applications (Venkatesh et al., 2003). Previous research has shown that PEU influences the motivation to use e-learning technologies as a sustainable resource in higher education (Chen and Tseng, 2012; Chow et al., 2012; Naveed et al., 2020). As a result, the greater the PEU of an e-learning system, the more certain the intention to use it is, and the more probable it will be used. Through PU, PEU is also projected to have an indirect impact on the desire to utilize e-learning as a source of sustainability in higher education (Chen and Tseng, 2012). As a result, PEU is projected to have an indirect impact on users' intentions *via* PU. As a result, the following hypotheses emerged from this study.

Hypothesis (H7): There is a strong link between PEU and PU.
Hypothesis (H8): There is a strong link between PEU and BIU.
Hypothesis (H9): There is a strong link between PEU and AUE.

### Perceived Usefulness

Users of twenty-first century IS are being pushed to adopt more current and consumer technologies that provide them with more flexibility as information quality is a major predictor of purpose (Pikkarainen et al., 2004). In reality, a person's willingness to use a particular e-learning system for their activities is decided by their evaluation of its utility (Alamri et al., 2020a). The PU of e-learning systems' use as sustainable in higher education has been demonstrated to have a significant positive impact on assumption (Chen and Tseng, 2012; Cheng et al., 2012; Chow et al., 2012; Islam, 2012; Alalwan et al., 2019; Alamri et al., 2020b; Al-Rahmi et al., 2021a,b). As a result, the greater the PU of the use of an e-learning system as a source of educational continuity, the more positive the desire to utilize it is and, therefore, the more likely it will be used. As a result, the following hypotheses emerged from this study.

Hypothesis (H10): There is a link between PU and BIU.
Hypotheses (H11): There is a substantial link between PU and AUE.

### Behavioral Intention to Use

Davis (1989) defines the strength of one's intention to engage in a given activity as “the strength of one's intention to engage in a particular action.” There is a favorable effect relationship between the BIU and AUE system in higher education, according to Alkhalaf et al. (2012) and Chow et al. (2012). While there is a distinction between intention to use and system use, Petter et al. (2008) point out that in their revised model, the performance model of e-learning systems did not distinguish. As a consequence (Venkatesh et al., 2003; Cifuentes-Faura et al., 2021), supports the positive relationship between BIU and AUE. As a consequence, the intention to use is anticipated to have a positive influence on AUE in this study. As a result, the following hypothesis emerged from this study.

Hypothesis (H12): There is a strong link between BIU and AUE.

### AUE System During the COVID-19 Pandemic

Actual system use is used as a metric in both DeLone and McLean's (2003) IS performance model and Davis's TAM (Kruchten, 2015). Petter et al. (2008) discovered that “usage” had a little relationship with the system's benefits in a thorough literature review research. The association between device usage and benefits has been demonstrated to be strong in previous studies (Chen and Tseng, 2012; Garcia-Smith and Effken, 2013). At the corporate level, it has been shown that the use of an e-learning system to provide training courses to employees has a large and favorable impact on the company's net benefits (Chen and Tseng, 2012). Other research (Kositanurit et al., 2006; Halawi et al., 2008; Al-Rahmi et al., 2017) obtained similar results. As a result, we anticipate that the use of this technique will provide students with additional benefits, such as enhanced awareness, time savings, and systematic learning management.

## Materials and Methods

### Study Design

Two specialists assessed the substance of the questionnaire. Before commencing the data collection, consent for research purposes was acquired from a public institution. The study's intended audience was undergraduate and postgraduate students. A questionnaire was produced for this study, and it was used to target the intended population. As a result, quantitative methods have been established to examine theoretical models and hypotheses, and this inquiry employed a quantitative analytical survey. Measurement items were created from the literature study and were designed to cover each step of the construction process. Many institutions throughout the world, including those in Saudi Arabia, have pushed for the use of e-learning platforms as a way to ensure the sustainability and profitability of higher education. As a result, the purpose of this research is to use empirical evidence to construct a model for measuring students' actions in terms of BIU and AUE. As a result, undergraduate and postgraduate students who used e-learning were included in the study's sample. For items (questions) relevant to the TAM dimensions, IS performance model constructs, and demographic variables, a five-point Likert scale was employed. A five-point Likert scale was utilized, with the options being (1) strongly disagree, (2) disagree, (3) neither agree nor disagree, (4) agree, and (5) strongly agree. Factor loadings were used to establish build validity, convergent validity, Cronbach's α, and converging validity for model goodness of fit, as recommended by Hair et al. (2017). As a result, they issued 20 surveys for a pilot test and 473 questionnaires for the final test in this study, with all factors confirmed to be acceptable. Cronbach's α, according to Hair et al. (2017), is a measure of internal consistency, or how closely a group of things are connected. It is regarded as a scale dependability metric. Cronbach's α was determined to be 0.881 in this study using standardized items. Table 1 shows the Cronbach's α reliability coefficient for the pilot and final test constructions; all variables were judged to be accurate and appropriate (for further details, see Table 1).

**Table 1:** Reliability test (pilot and final).

**No**.	**Factors**	**Code**	**Pilot test**	**Final test**
1	System quality	SYQ	0.725	0.893
2	Service quality	SEQ	0.734	0.875
3	Quality life	QoL	0.830	0.907
4	Perceived ease of use	PEU	0.726	0.895
5	Perceived usefulness	PU	0.832	0.909
6	Behavioral intention to use	BIU	0.886	0.914
7	Actual use E-learning system	AUE	0.801	0.921

### Data Collection and Participants

E-learning models are created for KFU and BU institutions. This strategy ensures that e-learning and remote education are delivered to the highest standards throughout Saudi Arabia and the Middle East. Thus, to offer distant education, KFU and BU must conceive, develop, and execute a full-fledged e-learning Model. This model is used by more than 150,000 students. As a result, when the institutions were closed due to the COVID-19 epidemic, this study was done online from February to April 2021. Prior to the primary data collection, a survey instrument was devised and confirmed to look for criteria that predicted student use of an e-learning system as a source of academic sustainability. In total, 481 questionnaires were distributed among students at both universities, and eight students who did not use online learning were found. Thus, there are still only 473 respondents who used online learning, who were then put into the Social Sciences Statistical Package (SPSS) program. This study looks at postgraduate and undergraduate students at Bisha University and King Faisal University who utilized the e-learning system during the COVID-19 pandemic.

Table 2 shows the data collected from participants. In total, 171 (36.2%) of the 473 useable surveys were from male respondents, whereas 302 came from female respondents (63.8%). In addition, the institution received 264 (55.8%) responses from Bisha University and 209 (44.2%) from King Faisal University. There were 283 undergraduate students (59.8%) and 190 postgraduate students (40.2%). Furthermore, 135 (28.5%) were aged between 18 and 21, 128 (27.1%) were aged between 22 and 25, 44 (9.3%) were aged between 26 and 29, 55 (11.6%) were aged between 30 and 33, and 111 (23.5%) were above 34. In total, 269 people (56.9%) are full-time students, while 204 people (43.1%) are part-time students. In total, 173 (36.6%) students came from the department of education, 42 (8.9%) from the faculty of science, 97 (20.5%) from the faculty of arts and humanities, 30 (6.3%) from the faculty of medical science, and 131 (27.7%) from the faculty of computer science. At the time of AUE, 321 (67.9%) had used e-learning for <5 years, 85 (18.0%) for schooling during the COVID-19 epidemic from 5 to 10 years, and 67 (14.2%) for more than 10 years. Finally, 324 (68.5%) used e-learning all the time, 141 (29.8%) used it occasionally, and 8 (1.7%) did not use it at all during the COVID-19 epidemic.

**Table 2:** Demographic information.

**Characteristics**		***N***	**%**	**Characteristics**		***N***	**%**
Gender	Male	171	36.2	University	Bisha University	264	55.8
	Female	302	63.8		King Faisal University	209	44.2
Faculties	Education	173	36.6	Age	More 34 years	111	23.5
	Science	42	8.9		30–33 years	55	11.6
	Art and humanities	97	20.5		26–29 years	44	9.3
	Medical science	30	6.3		22–25 years	128	27.1
	Computer science	131	27.7		18–21 years	135	28.5
Level of education	Undergraduate	283	59.8	Type of study	Full time	269	56.9
	Postgraduate	190	40.2		Part time	204	43.1
Time of use E-learning	less 5 years	321	67.9	Use E-learning	Always	324	68.5
	5–10 years	85	18.0		Some time	141	29.8
	More 10 years	67	14.2		Not' use	8	1.7

### Measurement Instruments and Procedure

The measurement scales' material validity was tested using the construction components used in previous research. The research questionnaire was divided into two sections: the basic demographic information (gender, age, educational level, and specialism) and the questionnaire items examining SYQ, SEQ, and QoL were adapted from Azeiteiro (2015). Davis (1989) was used for PEU and PU, Lin (2011) was used for action intent to use, and Venkatesh et al. (2012) and Al-Rahmi et al. (2015b) were used for the practical use of an e-learning system as education sustainability. All instruments were received from a trustworthy source. As a result, variables were evaluated by self-report using multi-item measures based on previous research.

### Data Analysis Variables

The data were examined with the most recent version of IBM's SPSS. Structural equation modeling (SEM) was also used to assess the data (SEM-AMOS). Construct validity evaluation, convergent validity analysis, and discriminant validity analysis, as well as structural modeling, were used to establish the validity and reliability of the measurement models (Hair et al., 2017).

## Results and Analysis

### Measurement Model

The conceptual model in this study was tested using covariance-based SEM (CB-SEM). The use of CB-SEM has various benefits. CB-SEM provides three main benefits over traditional multivariate approaches: (1) explicit measurement error evaluation; (2) estimate of latent (unobserved) variables using seen variables; and (3) model testing, which enables a structure to be imposed and the data fit to be confirmed. The measurement model and the structural model were used as methodological measures. The structural model examines how e-learning is used in digital learning theories, whereas the measurement model examines construct efficiency, validity, and overall model fit. All of the measures were evaluated on a five-point Likert scale, 1 representing “strongly disagree” and 5 representing “strongly agree.” The model's validity is validated by confirmatory factor analysis, and all items are included in Table 3.

**Table 3:** Measurement model, item loadings, build reliability, and convergent validity.

**Factor**	**Code**	**Questions**	**Loading**	**CR**	**AVE**	**CA**
System quality	SYQ1	E-learning system provides me with teaching materials that are easy to use.	0.881	0.908	0.670	0.893
	SYQ2	E-learning system provides me with teaching materials that fit with the learning objectives.	0.944			
	SYQ3	E-learning system provides me with sufficient teaching materials	0.761			
Service quality	SEQ1	Overall, the level of service quality I received from the E-learning system during the class was good.	0.792	0.899	0.630	0.875
	SEQ2	Overall, the level of service quality I received from the E-learning system during the class was excellent.	0.814			
	SEQ3	Overall, the level of service quality I received from the E-learning system during the class was high.	0.852			
	SEQ4	E-learning provides a proper online assistance and explanation.	0.843			
Quality life	QoL 1	E-learning system getting my course info will helps me creatively.	0.773	0.903	0.594	0.907
	QoL 2	The use of E-learning system saves me money and time.	0.732			
	QoL 3	The use of E-learning system provides more opportunities to participate in the class.	0.813			
	QoL 4	E-learning system communication channel with others saves me expense and effort.	0.711			
	QoL5	Overall, using the E-learning system help improving my quality of life.	0.800			
Perceived ease of use	PEU1	Learning to operate the E-learning system is easy for me.	0.762	0.885	0.662	0.895
	PEU2	I find it easy to get the E-learning system to do what I want it to do.	0.702			
	PEU3	My interaction with E-learning system is clear and understandable.	0.721			
	PEU4	It is easy for me to become skillful at using the E-learning system.	0.762			
	PEU5	I find the E-learning system easy to use.	0.803			
Perceived usefulness	PU1	Using the E-learning system can improve my academic performance.	0.761	0.923	0.606	0.909
	PU2	Using the E-learning system enables me to accomplish tasks more quickly.	0.824			
	PU3	Using the E-learning system increases my productivity.	0.792			
	PU4	Using the E-learning system can enhance my effectiveness.	0.802			
	PU5	Using the E-learning system makes it easier to learn course content.	0.791			
Behavioral intention to use	BIU1	I intend to use E-learning system during the semester.	0.823	0.821	0.573	0.914
	BIU2	I will return to E-learning system often.	0.814			
	BIU3	I will continue to use E-learning system.	0.894			
	BIU4	I intend to use E-learning system frequently for my study process.	0.864			
Actual use E-learning system	AUE1	On average, I use E-learning system less than two times a week.	0.774	0.893	0.681	0.921
	AUE2	On the average school day, spend more than 2 h on E-learning system.	0.703			
	AUE3	I get involved a lot with the E-learning system.	0.762			
	AUE4	I tend to use the E-learning system frequently.	0.814			
	AUE5	I use E-learning system frequently.	0.830			
	AUE6	I spend a lot of time exploring within the E-learning system.	0.792			

### Measures of Reliability, Validity, and Measurement Model

Table 4 illustrates that the SEM-AMOS measurement model for each concept has specific properties of reliability and validity. Using the human CFA and model fitness indicators from the measurement model, the structural model was used to calculate the strength of the connection route. The measurement components are listed in Table 2. The findings show that item dependability is typically high, with most of items exceeding the 0.70 criterion (Hair et al., 2017). The constructions' internal consistency was measured using composite reliability, which ranged from 0.821 to 0.923, above the cut-off value of 0.70 (Hair et al., 2017). The average variance extracted (AVE) for the components ranged from 0.573 to 0.681, indicating convergent validity above 0.50 (Hair et al., 2017). Researchers used cross-loading, the square root of AVE (Fornell and Larcker ratio), the average shared variance (ASV), and the maximum shared variance (MSV) tests to assess discriminant validity. The value of the diagonal is higher than the values of the accompanying row and column numbers (values are in bold in Table 4). It denotes a greater link between the building and other buildings. The MSV is lower than the ASV but higher than the average absolute variance (AVE) in Table 4. As a result, the measurement variables are unique and singular (see Table 4).

**Table 4:** Discriminant validity.

**Factors**	**Code**	**AVE**	**ASV**	**MSV**	**QoL**	**SEQ**	**SYQ**	**PEU**	**PU**	**BIU**	**AUE**
Quality life	QoL	0.594	0.021	0.072	0.889						
Service quality	SEQ	0.630	0.120	0.098	0.439	0.850					
System quality	SYQ	0.670	0.023	0.062	0.412	0.454	0.876				
Perceived ease of use	PEU	0.662	0.541	0.832	0.304	0.324	0.282	0.852			
Perceived usefulness	PU	0.606	0.643	0.101	0.357	0.342	0.318	0.277	0.851		
Behavioral intention to use	BIU	0.573	0.320	0.061	0.437	0.397	0.392	0.290	0.328	0.823	
Actual use E-learning system	AUE	0.681	0.192	0.084	0.328	0.302	0.294	0.300	0.272	0.346	0.887

### Model Fit Assessment

**Figure 2:**
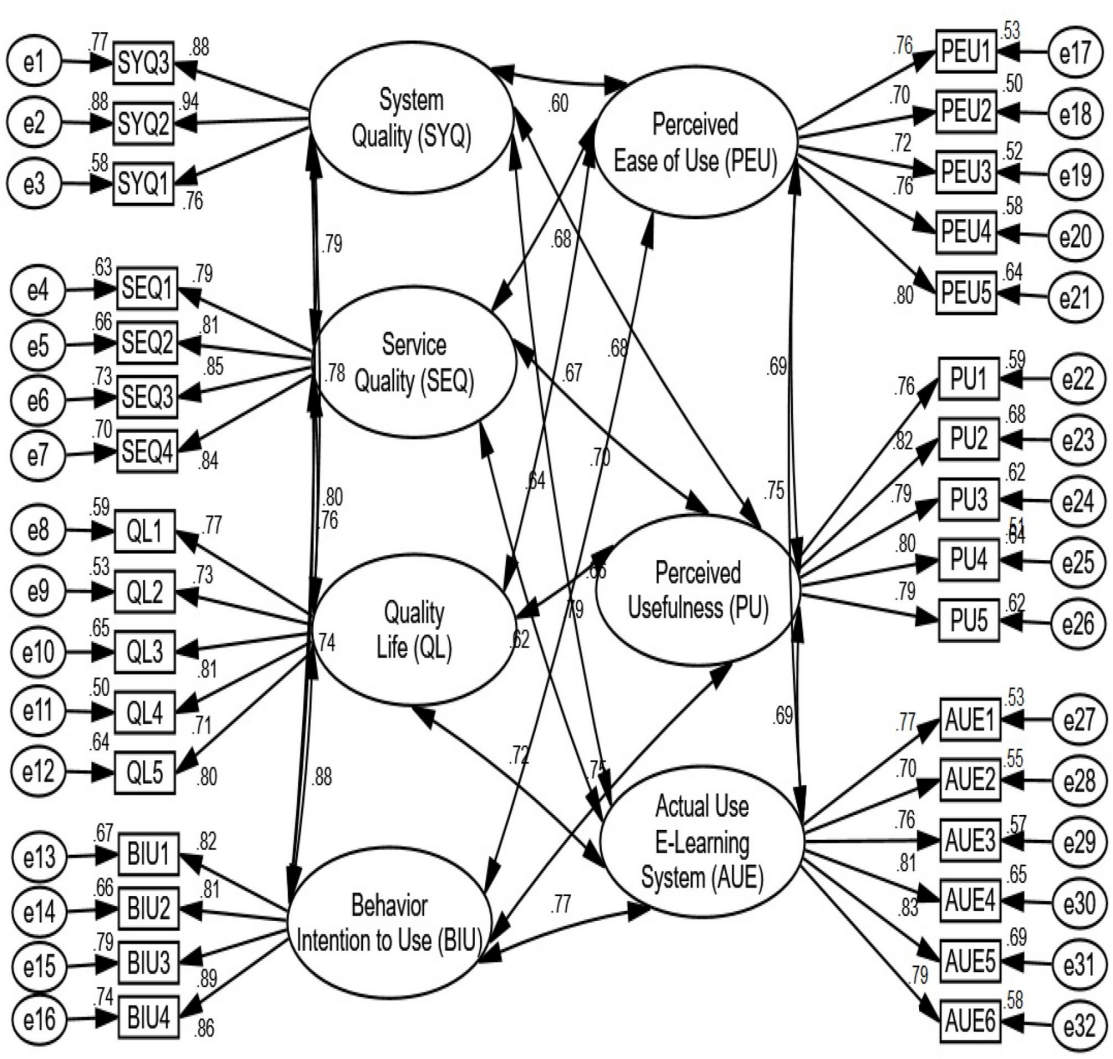
Measurement model.

Table 5 shows a CMN/DF ratio of 3.778, which is less than the threshold value of 5.00. The Incremental Fit Index (IFI) (0.950) is appropriate, the GFI (0.961) is reasonable, the CFI (0.946) is appropriate, and the TLI (0.938) is adequate. Root Mean Square Residual (RMR) and root mean square error of approximation (RMSEA) of 0.35 (0.05) and 0.041 (0.08), respectively, were less than the threshold, indicating a satisfactory model fit (Alamri et al., 2020a,b). All findings are shown in Figure 2, which demonstrate that the measurement model fitted the structural model well and was suitable for it.

**Table 5:** Results of hypotheses testing.

**Hypotheses and path**				**Beta (β)**	**SE**	**Critical ratio**	***P*-value**	**Results**
Hypothesis 1	PEU	< –	SYQ	0.113	0.050	2.241	0.025	Accepted
Hypothesis 2	PU	< –	SYQ	0.121	0.043	2.794	0.005	Accepted
Hypothesis 3	PEU	< –	SEQ	0.251	0.047	5.289	0.000	Accepted
Hypothesis 4	PU	< –	SEQ	0.105	0.042	2.504	0.012	Accepted
Hypothesis 5	PEU	< –	QoL	0.251	0.047	5.312	0.000	Accepted
Hypothesis 6	PU	< –	QoL	0.315	0.042	7.519	0.000	Accepted
Hypothesis 7	PU	< –	PEU	0.250	0.040	6.302	0.000	Accepted
Hypothesis 8	BIU	< –	PEU	0.314	0.044	7.148	0.000	Accepted
Hypothesis 9	AUE	< –	PEU	0.341	0.042	8.042	0.000	Accepted
Hypothesis 10	BIU	< –	PU	0.535	0.044	12.166	0.000	Accepted
Hypothesis 11	AUE	< –	PU	0.094	0.046	2.028	0.043	Accepted
Hypothesis 12	AUE	< –	BIU	0.414	0.042	9.795	0.000	Accepted

### Path Coefficient and Structural Model

**Figure 3:**
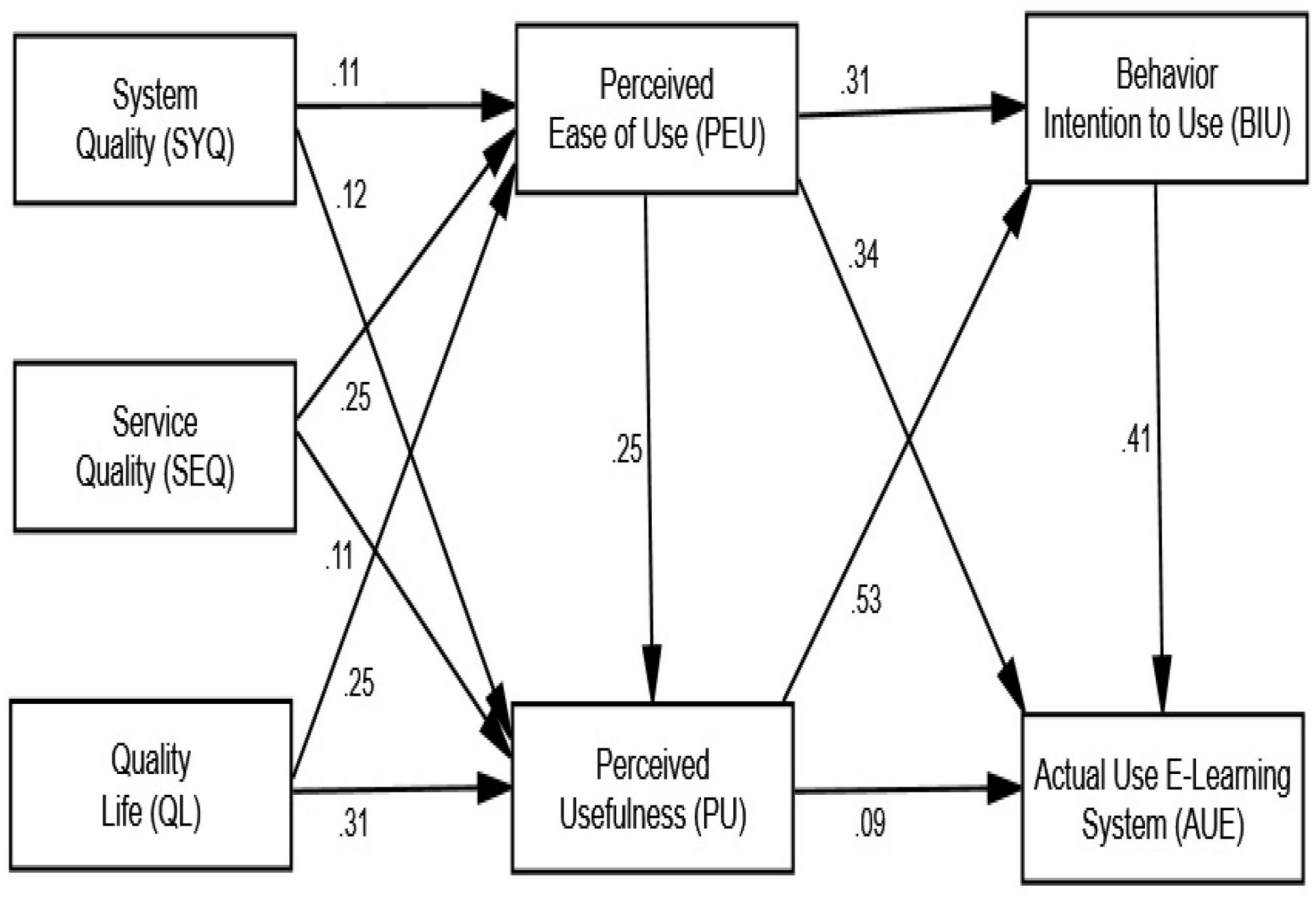
Structural model (*p*-value).

The structural model, as shown in Figure 3, defines the interaction and influence of independent factors on the dependent variable (path coefficient). Multiple connections, as well as moderating and mediating effects among multi-item variables, can be discovered using the SEM approach, particularly the maximum likelihood method (Berraies et al., 2017). The route coefficient depicts the direct influence of the latent predictor variable on predicted variables (see Figure 3). The goal of this study was to investigate and develop a new model for the use of an e-learning system as a method to ensure educational sustainability, based on a mixture of TAM and IS success models. As shown in Figure 3 and Table 5, the study contributes to the body of knowledge by giving empirical confirmation of the direct impact on learning for University students when utilizing an e-learning system as a long-term educational technique. Table 5 presents that all assumptions were supported, indicating that the use of an e-learning system as a long-term education model during the COVID-19 epidemic has a positive influence on TAM and IS success. This research also contributes to the present TAM and IS success model as sustainability in numerous ways by expanding the contributions of TAM (Davis, 1989) and the IS performance model (DeLone and McLean, 2003). TAM (Davis, 1989) and IS performance model (DeLone and McLean, 2003) are the most prominent theoretical contributions to the adoption analysis and are widely used by researchers to use e-learning systems as sustainability for education during the COVID-19 pandemic in Saudi Arabia. Table 5 presents that SYQ (β = 0.113, CR = 2.241, *p* < 0.001) has an important and positive impact on PEU, as stated in Hypothesis 1. Also, SYQ (β = 0.121, CR = 2.794, *p* < 0.001) has a positive and important impact on PU, as stated in Hypothesis 2. SEQ (β = 0.251, CR = 5.289, *p* < 0.001) has an important and positive impact on PEU, as stated in Hypothesis 3. Similarly, SEQ (β = 0.105, CR = 2.504, *p* < 0.001), as stated in Hypothesis 4, it has a positive and important impact on PU. Quality life (β = 0.251, CR = 5.312, *p* < 0.001), as stated in Hypothesis 5, is approved because it has a positive and important impact on PEU. As well, quality life (β = 0.315, CR = 7.519, *p* < 0.001) has a positive and significant effect on PU, as stated in Hypothesis 6. PEU (β = 0.250, CR = 6.302, *p* < 0.001) has a positive and significant effect on PU, as stated in Hypothesis 7. As well, PEU (β = 0.314, CR = 7.148, *p* < 0.001) has a positive and significant effect on BIU, as stated in Hypothesis 8, and PEU (β = 0.341, CR = 8.042, *p* < 0.001) has an important and positive impact on AUE, as stated in Hypothesis 9. PU (β = 0.535, CR = 12.166, *p* < 0.001) has a positive and significant effect on BIU which Hypothesis 10 accepted, and PU (β = 0.094, CR = 2.028, *p* < 0.001) has a major and positive impact on AUE as stated in Hypothesis 11. Finally, BIU (β = 0.414, CR = 9.795, *p* < 0.001) has a positive and significant effect on AUE as stated in Hypothesis 12 (see Figure 3 and Table 5).

### Description and Analysis of Factors

Standard deviation (SD) and mean are the two statistics that describe how measurements in a population deviate from the average (mean) or expected value. Data are grouped around the mean when the SD is low, while data are more spread out when the SD is large. An SD around 0 suggests that data points are close to the mean, whereas a high or low SD indicates that the data points are above or below the mean, respectively. Therefore, most of the data points are near to the mean when the SD is low. If the SD is high, the data are more dispersed. As a consequence, as shown in Tables 6–**12**, all values were adopted, meaning that the adoption of e-learning among University students increased academic performance “as a source of educational sustainability throughout the COVID-19 epidemic.” The following are the numerals' meanings: 1: “Strongly disagree;” 2: “Disagree;” 3: “Neutral;” 4: “Agree;” 5: “Strongly agree;” F: “Frequency;” %: “Percentages.” According to the data, the vast majority of students are in favor or strongly agree with SYQ, as well as PU and convenience of use. As a consequence, SYQ is defined in this study as the student's view that the adoption of an e-learning system as a long-term method of education throughout the COVID-19 epidemic will improve their education (see Table 6).

**Table 6:** Measuring system quality (SYQ).

**Factor and code**		**1**	**2**	**3**	**4**	**5**	**Mean**	**S.D**
		***F* (%)**	***F* (%)**	***F* (%)**	***F* (%)**	***F* (%)**		
System quality	SYQ1	4 (0.8%)	15 (3.2%)	50 (10.6%)	200 (42.3%)	204 (43.1%)	4.24	0.828
	SYQ2	4 (0.8%)	21 (4.4%)	62 (13.1%)	201 (42.5%)	185 (39.1%)	4.15	0.870
	SYQ3	5 (1.1%)	12 (2.5%)	59 (12.5%)	210 (44.4%)	187 (39.5%)	4.19	0.826

The final measurement consequences are shown in Table 7; the majority of students agree or strongly agree on SEQ, PU, and PEU. As a result, SEQ is defined in this study as the student's view that the use of e-learning as a long-term educational strategy during the COVID-19 epidemic would improve their learning (see Table 7).

**Table 7:** Measuring service quality (SEQ).

**Factors and code**		**1**	**2**	**3**	**4**	**5**	**Mean**	**S.D**
		***F* (%)**	***F* (%)**	***F* (%)**	***F* (%)**	***F* (%)**		
Service quality	SEQ1	11 (2.3%)	26 (5.5%)	60 (12.7%)	210 (44.4%)	166 (35.1%)	4.04	0.952
	SEQ2	9 (1.9%)	27 (5.7%)	72 (15.2%)	190 (40.2%)	175 (37.0%)	4.05	0.960
	SEQ3	8 (1.7%)	26 (5.5%)	75 (15.9%)	203 (42.9%)	161 (34.0%)	4.02	0.934
	SEQ4	6 (1.3%)	16 (3.4%)	78 (16.5%)	215 (45.5%)	158 (33.4%)	4.06	0.864

The final exam findings are shown in Table 8, with the vast majority of students agreeing or strongly agreeing with QoL, PU, and PEU. As a result, the QoL in this study is defined as the extent to which a student believes that the adoption of e-learning as a long-term educational strategy throughout the COVID-19 epidemic will improve their learning (see Table 8).

**Table 8:** Measuring quality life (QoL).

**Factor and code**		**1**	**2**	**3**	**4**	**5**	**Mean**	**S.D**
		***F* (%)**	***F* (%)**	***F* (%)**	***F* (%)**	***F* (%)**		
Quality life	QoL1	14 (3.0%)	14 (3.0%)	72 (15.2%)	186 (39.3%)	187 (39.5%)	4.10	0.962
	QoL2	9 (1.9%)	20 (4.2%)	62 (13.1%)	169 (35.7%)	213 (45.0%)	4.18	0.945
	QoL3	7 (1.5%)	12 (2.5%)	60 (12.7%)	192 (40.6%)	202 (42.7%)	4.21	0.865
	QoL4	7 (1.5%)	25 (5.3%)	63 (13.3%)	168 (35.5%)	210 (44.4%)	4.16	0.948
	QoL5	13 (2.7%)	22 (4.7%)	64 (13.5%)	185 (39.1%)	189 (40.0%)	4.09	0.981

The final measurement findings are shown in Table 9; the majority of students agree or strongly agree with PEU, PU, BIU, and AUE. As a consequence, PEU is defined in this study as the student's perception that the adoption of e-learning as a means of sustaining education during the COVID-19 epidemic is simple and beneficial to their learning (see Table 9).

**Table 9:** Measuring perceived ease of use (PEU).

**Factor and code**		**1**	**2**	**3**	**4**	**5**	**Mean**	**S.D**
		***F* (%)**	***F* (%)**	***F* (%)**	***F* (%)**	***F* (%)**		
Ease of use	PEU1	7 (1.5%)	8 (1.7%)	40 (8.5%)	205 (43.3%)	213 (45.0%)	4.29	0.809
	PEU2	6 (1.3%)	18 (3.8%)	73 (15.4%)	208 (44.0%)	168 (35.5%)	4.09	0.878
	PEU3	4 (0.8%)	15 (3.2%)	72 (15.2%)	200 (42.3%)	182 (38.5%)	4.14	0.849
	PEU4	3 (0.6%)	28 (5.9%)	48 (10.1%)	203 (42.9%)	191 (40.4%)	4.16	0.880
	PEU5	1 (0.2%)	23 (4.9%)	57 (12.1%)	191 (40.4%)	201 (42.5%)	4.20	0.849

The final measurement results are shown in Table 10; the majority of students agree or strongly agree with the PU of the e-learning system with BIU and AUE. As a consequence, PU is defined in this study as the student's conviction that the adoption of e-learning as a means of sustaining education during the COVID-19 epidemic is beneficial and would improve their learning (see Table 10).

**Table 10:** Measuring perceived usefulness (PU).

**Factor and code**		**1**	**2**	**3**	**4**	**5**	**Mean**	**S.D**
		***F* (%)**	***F* (%)**	***F* (%)**	***F* (%)**	***F* (%)**		
Perceived usefulness	PU1	2 (0.4%)	13 (2.7%)	42 (8.9%)	189 (40.0%)	227 (48.0%)	4.32	0.783
	PU2		10 (2.1%)	34 (7.2%)	195 (41.2%)	234 (49.5%)	4.38	0.712
	PU3	2 (0.4%)	15 (3.2%)	51 (10.8%)	191 (40.4%)	214 (45.2%)	4.27	0.809
	PU4	3 (0.6%)	21 (4.4%)	59 (12.5%)	189 (40.0%)	201 (42.5%)	4.19	0.865
	PU5	3 (0.6%)	18 (3.8%)	47 (9.9%)	190 (40.2%)	215 (45.5%)	4.26	0.835

The effects of the final measurement are shown in Table 11; the majority of students agree or strongly agree with their BIU and AUE. As a consequence, this study describes the extent to which a student feels that the use of e-learning systems as a long-term solution for education during the COVID-19 epidemic can improve their learning (see Table 11).

**Table 11:** Measuring behavioral intention to use (BIU).

**Factor and code**		**1**	**2**	**3**	**4**	**5**	**Mean**	**S.D**
		***F* (%)**	***F* (%)**	***F* (%)**	***F* (%)**	***F* (%)**		
Behavioral intention to use	BIU1	5 (1.1%)	11 (2.3%)	45 (9.5%)	197 (41.6%)	215 (45.5%)	4.28	0.813
	BIU2	8 (1.7%)	14 (3.0%)	49 (10.4%)	213 (45.0%)	189 (40.0%)	4.19	0.861
	BIU3	4 (0.8%)	10 (2.1%)	47 (9.9%)	225 (47.6%)	187 (39.5%)	4.23	0.778
	BIU4	4 (0.8%)	17 (3.6%)	43 (9.1%)	209 (44.2%)	200 (42.3%)	4.23	0.825

The majority of students are in favor or strongly agree with the practical application of the e-learning system as sustainability for education during the COVID-19 epidemic, as shown in Table 12. As a result, AUE is defined in this study as a student's belief that the use of an e-learning system as a sustainable instrument for education during the COVID-19 epidemic is simple and beneficial, and that it would enrich their learning (see Table 12).

**Table 12:** Measuring the actual use e-learning system (AUE) as sustainability for education during the COVID-19 pandemic.

**Factor and code**		**1**	**2**	**3**	**4**	**5**	**Mean**	**S.D**
		***F* (%)**	***F* (%)**	***F* (%)**	***F* (%)**	***F* (%)**		
Actual use E-learning	AUE1	5 (1.1%)	15 (3.2%)	38 (8.0%)	182 (38.5%)	233 (49.3%)	4.32	0.834
	AUE2	4 (0.8%)	23 (4.9%)	39 (8.2%)	167 (35.3%)	240 (50.7%)	4.30	0.878
	AUE3	4 (0.8%)	26 (5.5%)	86 (18.2%)	180 (38.1%)	177 (37.4%)	4.06	0.922
	AUE4	3 (0.6%)	25 (5.2%)	55 (11.6%)	193 (40.8%)	197 (41.6%)	4.18	0.879
	AUE5	6 (1.3%)	20 (4.2%)	56 (11.8%)	192 (40.6%)	199 (42.1%)	4.18	0.890
	AUE6	13 (2.7%)	48 (10.1%)	114 (24.1%)	166 (35.1%)	132 (27.9%)	3.75	1.056

## Discussion and Implications

The value-enhanced technology adoption (VETA) model was established by combining the components of the TAM and the IS success model to produce a new model, which we assessed in the context of the use of e-learning as a source of sustainability in Saudi higher education. The findings of this study add to the body of knowledge by indicating that students who enhance their e-learning system use it as a source of educational sustainability through SYQ, SEQ, and QoL. The research also contributes to the body of knowledge by establishing linkages between SYQ, SEQ, QoL, PEU, PU, BIU, and AUE values. Findings from the primary technological acceptance literature (Davis, 1989; Venkatesh et al., 2003, 2012) and past e-learning research (Al-rahmi et al., 2015a; Ching-Ter et al., 2017) support the degree and direction of the direct relationships between PEU, PU, BIU, and AUE.

By using second-order links in the TAM and IS success model, this work contributes to theory growth by bridging the gap between e-learning adoption research (Mohammadi, 2015; Abdullah and Ward, 2016; Alenazy et al., 2019) and the IS literature (Venkatesh et al., 2012). We observed that students' BIU has a positive impact on their AUE, and that independent variables SYQ, SEQ, and QoL had a positive impact on the mediator factors PU and PEU as a result of the research model. In fact, it indicates that the e-learning system has a more positive influence on students' faith in SYQ, SEQ, and QoL than other techniques. In their research, Mohammadi (2015) discovered that technical SYQ was the most important element in influencing AUE. They discovered that SYQ had a favorable influence on PEU, PU, and BIU in their research of e-learning systems as a source of educational sustainability, which fits with the findings of Rapley (2003) and Wu et al. (2012). The findings that PU has a significant impact on e-learning use intention (Chen and Tseng, 2012; Islam, 2012; Mohammadi, 2015; Al-Rahmi et al., 2019) have been verified. Despite the fact that it has been identified as a significant element in this relationship (Chen and Tseng, 2012; Islam, 2012; Mohammadi, 2015; Al-Rahmi et al., 2020), PEU was shown to have a little impact on the use of e-learning as a source of sustainability in higher education in this study. PU and PEU have direct and indirect effects on e-learning adoption, according to Sánchez et al. (2013). There is a need to increase digital sustainable development in higher education teaching, according to Sá and Serpa (2020), which implies considerable problems that higher education institutions must face and conquer if they are to be at the forefront of success in the worldwide education market. COVID-19 poses both obstacles and potential for higher education, and this paper explains both. As a result, students were unprepared for the shift, found it difficult to follow the course online, spent more time studying each day, and did worse in class (Faura-Martínez et al., 2021). Our findings show that PU has a considerable impact on students' behavioral intentions, whereas PEU has no direct impact, emphasizing the importance of usability in e-learning for education during the pandemic sustainability of COVID-19. PEU has an indirect influence on user intentions through PU, but it has no direct influence on user intentions. To put it another way, PU mediates the relationship between ease of use and user intentions, and the backdoor is the sole way to promote PEU against BIU and AUE. This study gave three empirical justifications. The empirical evidence of the use of e-learning system as education sustainability based on PEU and PU; the empirical proof of students' use actions and intentions; the empirical evidence of BIU and AUE through PEU and PU; the empirical evidence of PU and PEU e-learning system through SYQ, SEQ, and quality of life that can affect students' BIU and AUE; and the empirical evidence of PU and PEU e-learning system through SYQ, SEQ, and quality of life that can affect students' BIU. In summary, the following are the study's contributions:

- PEU and PU had a beneficial influence on students' behavioral desire to utilize e-learning and actual use of the e-learning system, according to the findings.
- Students' BIU e-learning and actual use of the e-learning system were the two most relevant factors in predicting e-learning use, according to the findings. PEU and PU were not significant determinants of behavioral intention, contrary to previous studies. The PEU and usefulness are influenced by SYQ, SEQ, and QoL. As a consequence, the research model contributed to the existing body of knowledge.
- Students' behavioral intention to use e-learning increases their actual use of the e-learning system. Lecturers and supervisors may also encourage students to use e-learning platforms by clearing up misunderstandings, sharing knowledge, and offering information to assist students improve their learning experiences, performance, and research skills.
- Students should be able to complement their classroom learning with the use of e-learning platforms.
- For e-learning systems used for educational and pandemic COVID-19 purposes, higher education institutions should study IS, SYQ, SEQ, and QoL. As a result, students' perceptions of the e-learning system's ease of use and perceived utility will influence their actual usage of an e-learning system as a source of educational sustainability during the COVID-19 pandemic.

## Conclusion, Limitations, and Future Perspectives

In this study, we utilized an integrated IS performance model and TAM to analyze students' perceptions of using the e-learning system as a source of educational sustainability, as well as the influence of SYQ, SEQ, and QoL on PEU and PU on students' BIU and AUE. In addition, the research model's interactions with the mediator components PE and PU positively influenced students' BIU, effect, and AUE. This research differs from previous research in the following ways: First, this study aims to combine the IS output model and the TAM into a unified model for e-learning as a source of educational sustainability. Second, unlike previous research in Saudi Arabia (Aljaber, 2018; Mutambik et al., 2020; Alqahtani et al., 2021), this study aims to provide a comprehensive review of recent publications in the field of e-learning as a source of long-term sustainability in higher education. Third, unlike previous research, this study focuses on the effects of variables on AUE by intention, as opposed to only looking at the effects of factors on intention to use. As a result, the current study is predicted to provide a wide range of outcomes and give crucial information about students' behavior, such as their BIU and AUE. According to our research, which was conducted at two public universities, SYQ, SEQ, and QoL had the most positive impact on BIU and AUE. Because demographic data, such as the impacts of age and gender, were not examined, it was not possible to conduct research on moderators. To analyze the effect of moderators on adoption in a broader study including many countries, institutions, or technologies, the researchers used the experimental power and data stability, as well as additional student satisfaction scores. This study has its own limits, regardless of the insights it provides. First, because this study focused on just two institutions, its conclusions should be taken with caution, as behavior at other universities (private universities, army universities, and other schools) may be different. Another drawback is the use of questionnaires to acquire qualitative data (interviews or observations). Because the data in this study were based on student viewpoints, which may differ from instructor judgments, variations in research fields were not taken into account. To overcome the study's limitations, future research might repeat the study in various countries and cultures. To investigate the similarities and contrasts between the many viewpoints of the unified theory of acceptance and use of technological variables according to context, a qualitative study would be appropriate. Further work is needed to adapt the findings to other circumstances, examine the model's breadth of applicability, and develop new applications following the construction and confirmation of TAM and IS success models in this study. In adapting the paradigm to socially relevant advances, these flaws must be kept in mind. The extension of the study to other technology-based areas, such as m-loyalty, e-organizational software adoption, and e-readiness, as well as a larger research sample, aims to increase the current understanding of the use of IS applications.

## Data Availability Statement

The original contributions presented in the study are included in the article/supplementary material, further inquiries can be directed to the corresponding author.

## Ethics Statement

Ethical review and approval was not required for the study on human participants in accordance with the local legislation and institutional requirements. Written informed consent from the patients/participants legal guardian/next of kin was not required to participate in this study in accordance with the national legislation and the institutional requirements.

## Author Contributions

AS, MAlq, MAla, and WA-R: conceptualization, methodology, investigation, resources, data curation, writing—original draft preparation, writing—review and editing, visualization, and supervision. MAla and WA-R: software. AS, MAlq, and WA-R: validation. MAlq and WA-R: formal analysis. AS and MAlq: project administration and funding acquisition. All authors have read and agreed to the published version of the manuscript.

## Funding

The authors extend their appreciation to the Deputyship for Research and Innovation, Ministry of Education of Saudi Arabia for funding this research work through the project number (UB-45-1442).

## Conflict of Interest

The authors declare that the research was conducted in the absence of any commercial or financial relationships that could be construed as a potential conflict of interest.

## Publisher's Note

All claims expressed in this article are solely those of the authors and do not necessarily represent those of their affiliated organizations, or those of the publisher, the editors and the reviewers. Any product that may be evaluated in this article, or claim that may be made by its manufacturer, is not guaranteed or endorsed by the publisher.

## Abbreviations

QoL: Quality Life
SEQ: Service Quality
SYQ: System Quality
PEU: Perceived Ease of Use
PU: Perceived Usefulness
BIU: Behavioral Intention to Use
AUE: Actual Use E-Learning System
TAM: Technology Acceptance Model
IS success model: Information System (IS) Success Model
SEM: Structural Equations Modeling.
